# Strengthened luteal phase support for patients with low serum progesterone on the day of frozen embryo transfer in artificial endometrial preparation cycles: a large-sample retrospective trial

**DOI:** 10.1186/s12958-021-00747-8

**Published:** 2021-04-23

**Authors:** Hongyuan Gao, Jing Ye, Hongjuan Ye, Qingqing Hong, Lihua Sun, Qiuju Chen

**Affiliations:** 1 Department of Assisted Reproduction, Shanghai Ninth People’s Hospital, Shanghai Jiaotong University School of Medicine, Shanghai, People’s Republic of China; 2 Centre of Assisted Reproduction, Shanghai East Hospital, Tongji University, Shanghai, People’s Republic of China

## Abstract

**Background:**

Low serum progesterone on the day of frozen embryo transfer (FET) is associated with diminished pregnancy rates in artificial endometrium preparation cycles, but there is no consensus on whether strengthened luteal phase support (LPS) benefits patients with low progesterone on the FET day in artificial cycles. This single-centre, large-sample retrospective trial was designed to investigate the contribution of strengthened LPS to pregnancy outcomes for groups with low progesterone levels on the FET day in artificial endometrium preparation cycles.

**Methods:**

Women who had undergone the first artificial endometrium preparation cycle after a freeze-all protocol in our clinic from 2016 to 2018 were classified into two groups depending on their serum progesterone levels on the FET day. Routine LPS was administered to group B (*P* ≥ 10.0 ng/ml on the FET day, *n* = 1261), and strengthened LPS (routine LPS+ im P 40 mg daily) was administered to group A (*P* < 10.0 ng/ml on the FET day, *n* = 1295). The primary endpoint was the live birth rate, and the secondary endpoints were clinical pregnancy, miscarriage and neonatal outcomes.

**Results:**

The results showed that the clinical pregnancy rate was significantly lower in group A than in group B (48.4% vs 53.2%, adjusted risk ratio (aRR) 0.81, 95% confidence interval (CI) 0.68, 0.96), whereas miscarriage rates were similar between the two groups (16.0% vs 14.7%, aRR 1.09, 95% CI 0.77, 1.54). The live birth rate was slightly lower in group A than in group B (39.5% vs 43.3%, aRR 0.84, 95% CI 0.70, 1.0). Birthweights and other neonatal outcomes were similar between the two groups (*P* > 0.05).

**Conclusions:**

The results indicated that the serum progesterone level on the FET day was one of the risk factors predicting the chances of pregnancy in artificial endometrium preparation cycles, and strengthened LPS in patients with low progesterone on the FET day might help to provide a reasonable pregnancy outcome in artificial cycles, although further prospective evidence is needed to confirm this possibility.

**Supplementary Information:**

The online version contains supplementary material available at 10.1186/s12958-021-00747-8.

## Introduction

Nearly 5 million babies resulting from assisted conception (in vitro fertilization (IVF)/intracytoplasmic sperm injection (ICSI)) have been delivered, and the demand for these fertility treatments is increasing. Meticulous ovarian stimulation and well-programmed luteal phase support (LPS) are the foundation of treatment success. Although the importance of LPS in IVF/ICSI cycles is well established, the optimal route, dose and duration of this support are still debated [1]. Artificial endometrial preparation for a frozen embryo transfer (FET) cycle is different from a stimulated IVF cycle in that there is no endogenous progesterone production; therefore, instead of luteal phase supplementation, there is a need for luteal phase “creation” or replacement [2]. In this context, artificial cycles provide us with a chance to explore the optimal dose and route of progesterone supplementation to support embryo implantation without the interferences of endogenous production in stimulation cycles [3].

Progesterone supplementation in artificial FET cycles can be provided by multiple routes, including the oral, intramuscular, vaginal and rectal routes. The oral route entails extensive first-pass metabolism, which limits its efficacy for luteal support. Vaginal administration is preferred to intramuscular injection due to its uterine first-pass effect, convenience and good tolerability [3]. However, body mass index (BMI) and the vaginal environment affect serum levels after vaginal administration, and serum progesterone concentrations show a marked inter-individual difference even when similar doses of progesterone are administered by the vaginal route [4]. Low serum progesterone levels on the FET day in artificial cycles using vaginal progesterone have been reported to be associated with compromised reproductive outcomes [5–7]; however, there is no consensus on whether strengthened LPS is beneficial for patients with low progesterone levels in artificial cycles [8, 9]. The debate over whether to use a one-size-fits-all regimen or individualized protocols in artificial endometrial preparation cycles is still active.

In our clinic, serum progesterone levels were routinely monitored in artificial endometrium preparation cycles. For patients receiving routine LPS and having low serum progesterone levels (< 10.0 ng/ml) on the FET day, we added intramuscular progesterone 40 mg daily to salvage the FET cycles. To investigate the contribution of strengthened LPS in women with low progesterone levels on the FET day, we performed a single-centre, large-sample retrospective trial to compare the pregnancy outcomes of artificial cycles between the two treatments.

### Study design and population

A retrospective study was conducted at the Department of Assisted Reproduction of the Ninth People’s Hospital of Shanghai Jiao Tong University School of Medicine. Women who had undergone the first artificial cycle for endometrium preparation in a freeze-all policy during the period from January 2016 to December 2018 were enrolled. The exclusion criteria were as follows: patients older than 42 years of age, a history of recurrent miscarriages or recurrent implantation failure (e.g., unsuccessful transfer of ≥3 times), systemic diseases, uterine diseases (e.g., fibroids and congenital uterine malformation) or hydrosalpinx. Cycles with missing information on serum hormones on the FET day were excluded. Only the first artificial cycle after the freeze-all protocol in our clinic was included for each patient, although some patients had previous FET failures before entering our clinic.

This study protocol was approved by the ethical committee of the hospital and was carried out in accordance with the Declaration of Helsinki. Due to the retrospective nature of the study, informed consent was not required, and patient data were used anonymously.

### Frozen embryo transfer and luteal phase support

For endometrium preparation in FET cycles, oral 17β-oestradiol (Fematon red tablets 4 mg, twice daily; Abbott Healthcare Products B.V.) or ethinyl oestradiol 25 μg three times daily commenced on the third day of a natural or progesterone withdrawal menstrual cycle. After 12–14 days, vaginal ultrasound examination was performed. When the endometrial thickness reached ≥7 mm, ultrasound detected quiescent ovaries, and the serum progesterone level was < 1.0 ng/ml, secretory transformation was initiated using Fematon yellow tablets (containing oestradiol 4 mg and dydrogesterone 20 mg twice daily, Abbott Healthcare Products B.V.) and vaginal micronized progesterone capsules 200 mg twice daily (Utrogestan, Laboratoires Besins International, France). Embryo transfer was performed 3 days after P administration for cleavage-stage embryos or 5 days later for blastocyst transfer.

All patients completed the serum hormone examination of progesterone and oestradiol at 8:00–9:00 on the transfer day, and the last dose before FET was administered on the morning of the FET day. The vitrified-warmed embryo transfers were arranged at 13:30–15:30. Based on the serum progesterone levels on the FET day, patients were classified into the two groups. For the cases with serum progesterone levels ≥10.0 ng/ml, the previously described luteal phase support was continued (Group B: normal P+ routine LPS). For patients with serum progesterone levels < 10.0 ng/ml, intramuscular progesterone 40 mg daily was added to strengthen luteal phase support after FET (Group A: low P +strengthened LPS). If the serum human chorionic gonadotropin (hCG) test was positive, LPS was continued until 10 weeks of gestation.

### Embryo quality assessment and vitrification

The ovarian stimulation regimen included the gonadotropin-releasing hormone (GnRH) antagonist protocol, GnRH agonist long protocol and progestin-primed ovarian stimulation. The embryo morphology assessment was evaluated on days 3, 5 and 6 after oocyte retrieval. Cleavage-stage embryos with at least 7 blastomeres and fragmentation < 20% were regarded as high-quality embryos. Blastocysts were scored according to the Gardner and Schoolcraft grading system [10] and recorded as high quality if they reached at least an expansion stage 3 with A or B for inner cell mass and trophectoderm (3BB). The vitrification and thawing procedure were previously described by Kuwayama et al. [11]. Briefly, embryo vitrification was carried out via a Cyrotop carrier system in combination with DMSO-EG-S as cryoprotectants. For thawing, embryos were transferred into dilution solution in a sequential manner.

Up to 2 embryos were transferred in FET cycles, and the selection of embryos was dependent on the order of embryo score. Briefly, the high-quality embryos were firstly transferred according to the order of embryo score; 2 embryos per transfer were preferred if possible. If there was only one cleavage embryo and one blastocyst, the blastocyst was transferred as priority.

### Outcome parameters and statistical methods

The primary outcome of the study was the live birth rate. The secondary endpoints included rates of implantation, biochemical pregnancy, clinical pregnancy, miscarriage and perinatal outcomes. Live birth was defined as a live neonate born after 24 weeks of gestation. A clinical pregnancy was confirmed by the observation of a gestational sac on ultrasound scanning 4–5 weeks after embryo transfer. The miscarriage rate was defined as a loss of clinical pregnancy before the 24th gestational week. The implantation rate was calculated as the number of gestational sacs visualized on ultrasound examination divided by the number of embryos transferred. All calculations were made on a per transfer cycle.

Low birth weight and macrosomia were defined as birth weights < 2500 g and ≥ 4000 g, respectively. Preterm birth was defined as a delivery before the completion of 37 gestational weeks. Pregnancy-related complications included gestational diabetes, intrahepatic cholestasis of pregnancy, pregnancy-induced hypertension and pre-eclampsia. All neonatal and delivery information was obtained from medical records or telephone interviews by a trained nurse.

The baseline characteristics and associated clinical outcomes were compared via *t*-test or chi-square test where appropriate. A multivariate logistic regression analysis was performed to determine the independent effect of serum progesterone on reproductive outcomes after adjustment for possible confounding factors, including maternal age, BMI (underweight, normal weight, overweight and obesity), infertility duration, gravidity, parity, infertility cause (tubal, polycystic ovary syndrome (PCOS), male, endometriosis and other), previous IVF failures, and number and stage of embryos transferred. We analysed the pregnancy outcomes for the groups to evaluate the effects of strengthened LPS for the group with low serum P on the FET day.

All statistical analyses were performed with the Statistical Package for Social Sciences (SPSS) version 21.0. A *P* value of < 0.05 was considered to be statistically significant.

## Results

A total of 7674 artificial endometrium preparation cycles were screened, and 2556 cycles met the inclusion criteria and underwent the first artificial cycle for FET during the study period. The mean age of the participating women was 31.3 ± 3.5 years, and their mean BMI was 21.76 ± 3.13 kg/m^2^. The median oestradiol levels on the FET day in artificial cycles were 210.0 pg/ml (interquartile range 116.0 pg/ml). The median P level on the FET day in artificial cycles was 9.9 ng/ml (interquartile range 4.2 ng/ml). A total of 1261 women (49.3%) with serum progesterone ≥10.0 ng/ml on the FET day received routine LPS (group B). A total of 1295 women (50.7%) were recorded with serum progesterone < 10.0 ng/ml on the FET day, then intramuscular progesterone 40 mg daily was added from the FET day onwards, and they were classified into group A.

**Fig. 1:**
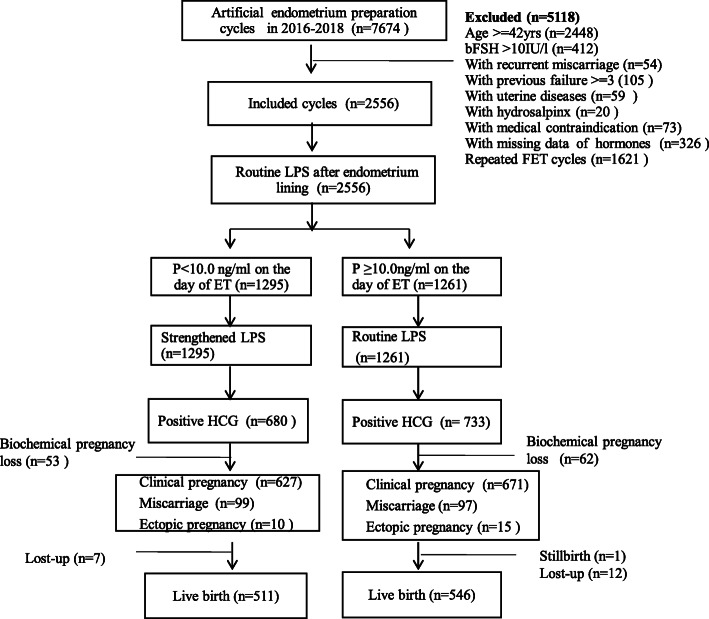
The flowchart of this study

A flow diagram of the patient selection process is shown in Fig. 1. Baseline characteristics between the two groups are presented in Table 1. Women in group A were similar to those in group B in terms of age, infertility duration, gravity, parity, endometrial thickness, and the number and stage of embryos transferred. In group A, the BMI and antral follicle counts (AFCs) were significantly higher (*P* < 0.05), and the basal follicle-stimulating hormone (FSH) levels were slightly lower than those in group B (*P* < 0.05). Compared with the control, women in group A more frequently experienced obesity (7.2% vs 4.6%), had a lower proportion of underweight individuals (9.4% vs 13.6%) and had a slightly higher proportion of individuals with PCOS (17.2% vs 13.6%, *P* < 0.05).

**Table 1:** The basic characteristics of the population

	Low P+ strengthened LPS (Group A, ***n*** = 1295)	Normal P+ routine LPS (Group B, ***n*** = 1261)	***P*** value
Age (years)	31.3 ± 3.5	31.4 ± 3.5	0.49
BMI (kg/m^2^)	22.11 ± 3.26	21.76 ± 3.13	0.005
Underweight (< 18.5 kg/m^2^)	121 (9.4%)	170 (13.6%)	
Normal weight (18.5–22.9 kg/m^2^)	733 (57.0%)	690 (55.2%)	
Overweight (22.9–27.4 kg/m^2^)	339 (26.4%)	332 (26.6%)	
Obesity (> 27.5 kg/m^2^)	92 (7.2%)	57 (4.6%)	
Infertility duration (yrs)	2.9 ± 2.7	2.9 ± 2.6	0.58
Basal FSH (mIU/ml)	5.61 ± 1.39	5.74 ± 1.43	0.017
Antral follicle counts	14.6 ± 7.7	13.6 ± 6.8	0.002
Gravidity			0.072
0	735 (56.8%)	671 (53.2%)	
≥ 1	560 (43.2%)	590 (46.8%)	
Parity			0.41
0	1216 (93.9%)	1174 (93.1%)	
≥ 1	79 (6.1%)	87 (6.9%)	
Infertility causes			0.12
Tubal	735 (56.8%)	768 (60.9%)	
PCOS	223 (17.2%)	172 (13.6%)	
Male	124 (9.6%)	118 (9.4%)	
Endometriosis	36 (2.8%)	35 (2.8%)	
Other	177 (13.7%)	168 (13.3%)	
Previous IVF failures			0.43
0	1114 (86.2%)	1105 (87.5%)	
1–2	67 (5.2%)	53 (4.2%)	
≥ 3	114 (8.8%)	103 (8.2%)	
Ovarian stimulation			0.19
GnRHa long protocol	105 (8.1%)	118 (9.4%)	
GnRH antagonist	214 (16.5%)	180 (14.3%)	
PPOS	976 (75.4%)	963 (76.4%)	
Serum estrogen on FET day (pg/ml) median (IQR)	203.0 (112.0)	216.0 (123.5)	0.082
Serum P on FET day (ng/ml) median (IQR)	6.8 (2.3)	12.2 (3.4)	< 0.001
Endometrium thickness (mm)	9.82 ± 1.84	9.82 ± 1.88	0.99
Number of embryos transferred			0.063
Single	306 (23.6%)	254 (20.1%)	
Double	989 (76.4%)	1007 (79.8%)	
Embryo stage			0.11
Cleavage	1079 (83.3%)	1020 (80.9%)	
Blastocyst	216 (16.7%)	241 (19.1%)	

A multinomial logistic analysis to predict serum progesterone < 10.0 ng/ml on the FET day, with all meaningful clinical parameters included (age, BMI, basal FSH levels and infertility causes), showed that only BMI was negatively related with serum progesterone on the FET day in artificial cycles (*P* < 0.05), mainly the subgroup of underweight (BMI < 18.5 kg/m^2^) had a lower risk for low progesterone (risk ratio (RR) 0.68, 95% confidence interval (CI) 0.52, 0.88) while subgroup of obesity (BMI > 27.5 kg/m^2^) did not reach the significance of increased risk for low progesterone (RR 1.36, 95%CI 0.95,1.94).

### Implantation and pregnancy outcome

Pregnancy outcomes in the two groups are shown in Table 2. The clinical pregnancy rate in group A was lower than that in group B (48.4% vs 53.2%, adjusted RR 0.81, 95% CI 0.68–0.96), with a difference between groups of 4.8%. The implantation rates were 35.11 and 36.8% in groups A and B, respectively (crude RR 0.93, 95% CI 0.82–1.05, *P* = 0.051). The miscarriage rate in group A was similar to that in group B (16.0% vs 14.7%, adjusted RR 1.09, 95% CI 0.77–1.54). The live birth rate in group A was slightly lower than that in group B (39.5% vs 43.3%, adjusted RR 0.84, 95% CI 0.70–1.0).

**Table 2:** The pregnancy outcomes in artificial FET cycles using strengthened luteal phase support

	Low P+ strengthened LPS (Group A, n = 1295)	Normal P+ routine LPS (Group B, n = 1261)	Crude RR (95%CI)	Adjusted RR (95%CI)	*P* value
Biochemical pregnancy rate	52.5% (680/1295)	58.1% (733/1261)	0.80 (0.68,0.93)	0.80 (0.67.0.96)	0.014
Clinical pregnancy rate	48.4% (627/1295)	53.2% (671/1261)	0.91 (0.84,0.98)	0.81 (0.68,0.96)	0.016
Multiple pregnancy rate	24.9% (186/627)	26.7% (179/671)	0.96 (0.85,1.07)	0.79 (0.58,1.09)	0.15
Miscarriage rate	16.0% (99/627)	14.7% (97/671)	1.05 (0.90,1.22)	1.09 (0.77,1.54)	0.62
Live birth rate	39.5% (511/1295)	43.3% (546/1261)	0.92 (0.85.1.0)	0.84 (0.70,1.0)	0.054

Adjusted for BMI, baseline FSH values, antral follicle counts, the number and stage of embryos transferred

The comparison of neonatal birthweight was similar between the two groups, both for singleton births (3366.4 ± 536.0 g vs 3317.0 ± 516.8 g, *P* > 0.05) and for twin births (2461.0 ± 460.1 g vs 2472.6 ± 472.2 g, *P* > 0.05) (Table 3). The proportion of pregnancy-related complications and other neonatal outcomes, including the prevalence of preterm birth and low birthweight, were similar between the two treatment groups (*P* > 0.05) (Table 3).

**Table 3:** The neonatal outcome of artificial FET cycles using strengthened luteal phase support

Characteristics	Low P+ strengthened LPS (*n* = 511)	normal P+ routine LPS (*n* = 546)	*P* value
Singletons	363	412	
Newborn gender			0.342
Female	182	192	
Male	181	220	
Birthweight (g)	3366.4 ± 536.0	3317.0 ± 516.7	0.193
Gestation age (weeks)	38.5 ± 1.7	38.4 ± 1.9	0.060
Low birthweight (< 2500 g) n (%)	16 (4.4%)	18 (4.4%)	0.986
Macrosomia (≥4000 g) n (%)	38 (7.4%)	32 (7.8%)	0.195
Pregnancy-related complications n (%)	16 (4.4%)	32 (7.8%)	0.052
Twins	148^a^	134	
Newborn gender			0.563
Female	137	131	
Male	158	137	
Birthweight (g)	2461.0 ± 460.1	2472.6 ± 472.2	0.833
Gestation age (weeks)	35.6 ± 2.4	35.5 ± 2.5	0.087
Very low birthweight (< 1500 g) n (%)	16 (5.4%)	14 (5.2%)	0.891
Low birthweight (< 2500 g) n (%)	116 (39.3%)	100 (37.3%)	0.552
Pregnancy-related complications n (%)	25 (16.9%)	31 (23.1%)	0.212

^a^One boy of twins was stillbirth and the other lived birth

The subgroup analysis was performed according to the number and stage of embryos transferred (Table 4). In the subgroup of cleavage embryo transfer, the clinical pregnancy rate (47.5% vs 51.7%, aRR 0.79, 95% CI 0.65,0.96) and live birth rate in group A (38.6% vs 42.1%, aRR 0.82, 95%CI 0.67, 0.99) were lower than the controls, while the subgroup of blastocyst transfer was similar between the two groups. In the subgroup of single embryo transfer, the clinical pregnancy rate (36.3% vs 49.6%, aRR 0.57, 95% CI 0.38,0.84) and live birth rate in group A (28.4% vs 40.6%, aRR 0.56, 95%CI 0.38, 0.82) were significantly lower than that in group B, while the subgroups of two-embryo transfer showed the similar results between the two groups. The results demonstrated that the pregnancy potential in the FET cycles with cleavage embryo transfer and single embryo transfer was more obvious impacted by serum low progesterone on the FET day.

**Table 4:** The pregnancy outcomes of artificial FET cycles in the subgroups of different embryos

	Low P+ strengthened LPS (Group A, n = 1295)	Normal P+ routine LPS (Group B, n = 1261)	Crude RR (95%CI)	Adjusted RR (95%CI)	*P* value
**Cleavage embryo transfer**					
Clinical pregnancy rate	47.5% (513/1079)	51.7% (527/1020)	0.85 (0.71,1.0)	0.79 (0.65,0.96)	0.017
Miscarriage rate	16.1% (82/508)	14.1% (73/517)	1.17 (0.83,1.65)	1.19 (0.81,1.76)	0.37
Live birth rate	38.6% (416/1079)	42.1% (429/1020)	0.86 (0.73,1.03)	0.82 (0.67,0.99)	0.044
**Blastocyst transfer**					
Clinical pregnancy rate	52.8% (114/216)	59.8% (144/241)	0.78 (0.52,1.16)	0.82 (0.54,1.24)	0.34
Miscarriage rate	15.2% (17/112)	17.0% (24/141)	0.87 (0.44,1.72)	0.82 (0.39,1.70)	0.82
Live birth rate	44.0% (95/216)	48.5% (102/241)	0.83 (0.58,1.20)	0.90 (0.60,1.36)	0.62
**Single embryo transfer**					
Clinical pregnancy rate	36.3% (111/306)	49.6% (126/254)	0.58 (0.41,0.81)	0.57 (0.38,0.84)	0.005
Miscarriage rate	20.9% (23/110)	17.6% (22/125)	1.24 (0.65,2.37)	1.25 (0.59,2.66)	0.56
Live birth rate	28.4% (87/306)	40.6% (103/254)	0.58 (0.41,0.83)	0.56 (0.38,0.82)	0.005
**Two-embryos transfer**					
Clinical pregnancy rate	52.2% (516/989)	54.2% (545/1006)	0.92 (0.77,1.10)	0.89 (0.73,1.08)	0.25
Miscarriage rate	14.9% (76/510)	14.1% (75/533)	1.07 (0.76,1.51)	1.08 (0.73,1.58)	0.71
Live birth rate	42.9% (424/989)	44.0% (443/1006)	0.95 (0.80,1.14)	0.93 (0.76,1.13)	0.45

Adjusted for BMI, baseline FSH values, antral follicle counts

**Table 5:** The independent predictors of pregnancy outcomes in artificial FET cycles by logistic regression analysis

	Clinical pregnancy				Live birth			
	RR	95% CI		*P* value	RR	95% CI		*P* value
**Female age**	0.96	0.94	0.99	0.00	0.96	0.93	0.98	0.00
**Infertility duration**	1.00	0.96	1.03	0.93	0.99	0.95	1.02	0.42
**Basal FSH value**	0.94	0.88	1.00	0.05	0.94	0.88	1.00	0.06
**Antral follicle counts**	1.01	0.99	1.02	0.46	1.00	0.99	1.01	0.95
**Embryo numbers** ^a^	1.92	1.52	2.43	0.00	1.80	1.42	2.29	0.00
**Embryo stage** ^b^	1.69	1.32	2.17	0.00	1.52	1.19	1.95	0.00
**Serum P on FET day** ^**c**^	0.81	0.68	0.96	0.02	0.84	0.70	1.00	0.05
**BMI** ^d^				0.77				0.81
**Underweight**	0.86	0.65	1.15	0.30	0.93	0.70	1.24	0.62
**Overweight**	1.00	0.81	1.24	0.97	0.91	0.74	1.13	0.40
**Obesity**	0.99	0.67	1.47	0.97	1.04	0.71	1.54	0.83
**Infertility causes** ^e^				0.01				0.15
**PCOS**	1.55	1.18	2.03	0.00	1.26	0.96	1.64	0.09
**Male**	1.41	1.04	1.92	0.03	1.38	1.02	1.87	0.04
**Endometriosis**	0.86	0.48	1.53	0.61	1.06	0.59	1.89	0.85
**Other**	1.08	0.83	1.40	0.56	1.21	0.93	1.57	0.16

^a^ Two- embryos transfer as reference group; ^b^ Cleavage embryo transfer as reference group; ^c^ serum P levels on the FET day ≥10.0 ng/ml as reference; ^d^ Normal weight as reference; ^e^ Tubal factor as the reference;

In order to eliminate the possible confounder of IVF failures, the patients without previous IVF failure were collected and the basic characteristics were shown in the supplemental Table 1. The clinical pregnancy rate in the subgroup of low P was significantly lower than that in the control (50.2%% vs 54.8%, aRR 0.83, 95%CI 0.67,0.98), and the live birth rate was slightly lower but did not reach the difference compared to the controls. The data of patients without IVF failures showed the same change trend as the mentioned population (supplemental Table 2).

### Low serum progesterone on the FET day was a risk factor for the chance of pregnancy

The risk factors associated with pregnancy outcomes were explored by logistic regression analysis (Table 5). When clinical pregnancy was chosen as the dependent factor and age, BMI (categorical variable), number of embryos transferred, embryo stage, infertility causes and serum progesterone levels (categorical variable) were chosen as independent factors, the variants of age, the number and stage of embryos, infertility causes and progesterone levels on the FET day were found to be significant independent prognosticators (*P* < 0.05). The age and number of transferred embryos had a negative influence on the pregnancy outcome, and the infertility causes of PCOS and male factor had a higher chance of pregnancy than the tubal factor in this study (*P* < 0.05). The low progesterone on the FET day (*P* < 10.0 ng/ml), in the context of strengthened LPS, still decreased the chance of clinical pregnancy by 19% after adjusting for confounding factors.

Multinomial logistic analysis, performed to predict the live birth rate, showed that only age, number and stage of embryos transferred were significantly related to the live birth rate (*P* < 0.05). Low serum progesterone values on the FET day slightly reduced the live birth rate but did not reach significance (aRR 0.84, 95% CI 0.70–1.0).

## Discussion

Clinicians frequently prefer artificial endometrial preparation because it facilitates the programming of embryo transfer [3]. The increasing number of artificial cycles raises the question of the serum progesterone levels required to optimize the pregnancy outcome. In this large-sample retrospective study, we investigated the contribution of strengthened LPS to pregnancy outcomes in patients with low serum progesterone on the FET day in artificial cycles. The strengthened LPS in the groups of low serum progesterone produced 39.5% live birth rate, a slightly lower (3.8%) than the groups with normal serum progesterone levels and usage of routine LPS.

The principles for choosing LPS include the minimum effective dose, good safety and tolerability [12]. This is an open question with many alternative answers, and the heterogeneous applications of the dose and routes make it difficult to compare LPS in different studies [3, 13]. Pregnancy outcomes, especially live birth rate, become the primary endpoint of evaluating the effects of LPS in artificial FET cycles. Vaginal administration was the first choice of doctors, used alone or in combination with oral or intramuscular injection in a recent large survey [14]. In our centre, the most popular regimen of LPS was using 20 mg oral dydrogesterone twice daily and 200 mg vaginal micronized progesterone twice daily in artificial cycles. In this context, the serum progesterone level was a surrogate marker reflecting the systematic absorption extent of vaginal progesterone administration. It might be interfered with by individual variability, such as BMI, mucus surface area, amount of cervical mucus and the difference in the vaginal microbiome [4]. Thus, a marked inter-individual difference in serum progesterone concentrations in the luteal phase was present despite the administration of the same dose of vaginal progesterone. The possibility of LPS insufficiency by the vaginal route should be considered, and sections of patients with low serum progesterone concentrations should be considered in artificial cycles. The previous report compared three arms in artificial cycles using a randomized controlled trial (vaginal 200 mg twice daily, im P 50 mg daily, or vaginal 200 mg twice daily+im P 50 mg every third day), the group receiving vaginal progesterone only was found to have a significantly lower ongoing pregnancy rate compared the other two groups (31% vs 50% vs 47%), and the trial did not continue after inter analysis for the higher proportion of biochemical pregnancy loss and miscarriage [15]. Although no data on serum progesterone levels in the three groups were available from that study, the low serum progesterone levels on the FET day in artificial cycles using the vaginal route were previously reported to be associated with poorer reproductive outcomes, and the cut-off value varied in previous reports (5.0–12.0 ng/ml) [5–8]. In this study, the cut-off value was set as 10.0 ng/ml.

In this study, strengthened P replacement was a protective approach, with an attempt to mitigate the effects of low serum progesterone that fell below this threshold. For the topic, the ideal control was using routine LPS for patients with low serum progesterone on the FET day, but our previous tendency of doctors was to add progesterone to avoid possible harm and maximize the patients’ benefits in a conservative view. Limited by the real-world data in our clinic, we converted to use the population of normal serum progesterone and routine LPS as controls. The current data showed slightly lower pregnancy outcomes of strengthened LPS in the lower progesterone group. Although our study cannot distinguish the difference origin from the harm caused by lower serum progesterone or the benefits of additional progesterone, the current comparison data still provided some meaningful information. First, the patients with low serum progesterone and strengthened LPS showed a slightly lower clinical pregnancy and live birth rate than the normal controls, which decreased the chance of clinical pregnancy by 19% after adjusting for confounding factors. Similar results were reported by Alsbjerg et al. [16]; serum progesterone levels below 11.0 ng/ml decreased the chance of ongoing pregnancy with a risk reduction of 14% in artificial FET cycles. Using the logistic regression model in this study, the low serum progesterone level, as a categorical variable, was a risk factor for the chance of pregnancy in artificial endometrial preparation cycles, which indicated the suboptimal condition in this section of patients with low serum progesterone.

Second, the pregnancy outcome of the groups with low progesterone and strengthened LPS was an acceptable result, and the clinical pregnancy rate (48.4%) and live birth rate (39.5%) appeared reasonable. A similar proportion of biochemical pregnancy loss and miscarriage also confirmed the efficacy of strengthened LPS. As such, even though serum progesterone was inadequate on the FET day, this was potentially remedied by additional progesterone administration, reinforcing intervention might still be possible beyond the day of transfer. The results were in coincide with the recent report of Volovsky et al. Progesterone replacement enhanced the pregnancy outcome if the progesterone on the FET day was lower than 8.0 ng/ml [8]. In a recent study by Polats et al., intramuscular progesterone supplementation every third day did not increase the ongoing pregnancy rate compared with vaginal progesterone only in vitrified blastocyst transfer cycles (48.3% vs 51.8%). However, the patients with serum progesterone less than 8.75 ng/ml among those receiving only vaginal progesterone had a numerically lower ongoing pregnancy rate (28.6% vs 46.6%) but did not reach a statistically significant difference [9].

In addition, the clinical pregnancy rate in the low progesterone group was decreased while the implantation rate did not reach the significance, the same trends were found in the population of patients without previous IVF failure. We further analysed the possible confounders of embryo factor. The sensitivity analysis showed that the clinical pregnancy rate and live birth rate showed a decreasing trend in the subgroups of cleavage embryo and in the subgroup of single embryo transfer, so we presumed that the endometrium in the context of low progesterone and strengthened LPS was prone to negatively impact on pregnancy potential in the FET cycles with cleavage embryo or single embryo transfer.

The current study also had several strengths. Vaginal progesterone administration achieved higher endometrial tissue concentrations and lower systemic exposures than those observed after intramuscular injection, and the dose-effect relationship was not obvious in the same route of progesterone administration [4]. Therefore, the combination of two or three routes, rather than increasing the dose of a single route, was a reasonable choice to meet the requirement of endometrial transfection in artificial cycles. Multiple routes of progesterone administration provided sufficient luteal support in artificial FET cycles. The secondary strength was the large cohort size, and it had sufficient power to answer the question of whether it was beneficial to strengthen LPS to overcome the possible negative influence in the cycles with low serum progesterone on the FET day. All cycle data were derived from a single institution, where consistency of practice could be assured.

Our study was limited by its retrospective design. In this context, we screened the database with strict inclusion criteria, analysis was restricted to first artificial cycles after the freeze-all protocol, and a number of potential confounders were well controlled in the current study. Additionally, the vast majority of patients in the present study were young women with normal ovarian reserve, therefore, the extrapolation to an unselected population needs to be validated.

In a previous report by Yovich et al., maternal age, embryo quality and mid-luteal serum progesterone levels were listed as three important factors governing the implantation rates for artificial FET cycles [17]. In this study, serum progesterone levels on the FET day, as a categorical variable, were found to be a significant independent prognosticator of clinical pregnancy, while it did not reach statistical significance for the live birth rate. The explanation of the results should be cautious. Strengthening LPS might be useful for patients with low serum progesterone levels on the FET day in artificial cycles. Further study is needed to perform randomized controlled trials to evaluate the individualization of progesterone dosages.

## Conclusions

In summary, our study confirmed that serum progesterone levels on the FET day might be one of the risk factors predicting the chance of pregnancy in artificial cycles, and careful monitoring of serum progesterone concentration was warranted. Strengthened LPS in patients with low progesterone on the FET day might help to achieve favourable pregnancy outcomes from artificial cycles, although the rate of pregnancy was slightly lower in that group than in patients with normal serum progesterone levels on the FET day and usage of routine LPS. Our study revealed new information on the topic of luteal phase support. Further studies should explore progesterone administration to optimize concentrations for individual women to improve pregnancy outcomes.

## Supplementary Information

**Additional file 1: Supplemental Table 1** The basic characteristics of the population without IVF failure.

**Additional file 2: Supplemental Table 2** The pregnancy outcomes of artificial FET cycles in women without IVF failures.

## Data Availability

The data is not publicly shared and please contact author for data requests.

## Abbreviations

IVF: In vitro fertilization
ICSI: Intracytoplastic sperm injection
FET: Frozen embryo transfer
LPS: Luteal phase support
BMI: Body mass index
hCG: Human chorionic gonadotropin
RR: Risk ratio
FSH: Follicle stimulation hormone
