# Supplementary material for: Strengthened luteal phase support for patients with low serum progesterone on the day of frozen embryo transfer in artificial endometrial preparation cycles: a large-sample retrospective trial
Source: Reprod Biol Endocrinol. 2021 Apr 23;19:60. doi: 10.1186/s12958-021-00747-8 (PMC8063468; doi:10.1186/s12958-021-00747-8)
Supplement: Supplementary file 1 — Additional file 1: Supplemental Table 1 The basic characteristics of the population without IVF failure. [file 12958_2021_747_MOESM1_ESM.docx]

**Supplemental table 1 The basic characteristics of the population without IVF failure**

|  | **Low P+ strengthened LPS**  **(Group A, n=1114)** | **Normal P+ routine LPS**  **(Group B, n=1105)** | **P value** |
| --- | --- | --- | --- |
| Age (years) | 31.0±3.5 | 31.2±3.5 | 0.20 |
| BMI (kg/m^2^) |  |  | <0.01 |
| Underweight (<18.5 kg/m^2^) | 106(9.6%) | 154(14.0%) |  |
| Normal weight (18.5-22.9 kg/m^2^) | 643 (58.0%) | 603(55.0%) |  |
| Overweight (22.9-27.4 kg/m^2^) | 273(24.6%) | 295(26.9%) |  |
| Obesity (>27.5 kg/m^2^) | 86(7.8%) | 45(4.1%) |  |
| Infertility duration (yrs) | 2.8±2.6 | 2.8±2.5 | 0.93 |
| Basal FSH (mIU/ml) | 5.58±1.37 | 5.72±1.43 | 0.016 |
| Antral follicle counts | 14.9±7.7 | 13.9±6.9 | 0.005 |
| Gravidity |  |  | 0.11 |
| 0 | 638(57.3%) | 596(53.9%) |  |
| ≥1 | 476(42.7%) | 609(46.1%) |  |
| Parity |  |  | 0.52 |
| 0 | 1041(93.4%) | 1025(92.8%) |  |
| ≥1 | 73(6.6%) | 80(7.3%) |  |
| Infertility causes |  |  | 0.059 |
| Tubal | 613(55.0%) | 663(60.0%) |  |
| PCOS | 211(18.9%) | 163(14.8%) |  |
| Male | 104(9.3%) | 106(9.6%) |  |
| Endometriosis | 32(2.9%) | 25(2.3%) |  |
| Other | 154(13.8%) | 148(13.4%) |  |
| Ovarian stimulation |  |  | 0.23 |
| GnRHa long protocol | 101(9.1%) | 109(9.9%) |  |
| GnRH antagonist | 203(18.2%) | 172(15.6%) |  |
| PPOS | 810(72.7%) | 824(74.6%) |  |
| Serum estrogen on FET day (pg/ml)  median (IQR) | 202.0 (114.0) | 215.0(122.0) | <0.01 |
| Serum P on FET day (ng/ml)  median (IQR) | 8.0(2.1) | 12.2(3.4) | <0.01 |
| Endometrium thickness (mm) | 9.59±2.34 | 9.66±2.23 | 0.45 |
| Number of embryos transferred |  |  | 0.039 |
| Single | 261(23.4%) | 219(19.8%) |  |
| Double | 853(76.6%) | 886(80.2%) |  |
| Embryo stage |  |  | 0.13 |
| Cleavage | 926(83.1%) | 891(80.6%) |  |
| Blastocyst | 188(16.9%) | 214(19.4%) |  |
