# Supplementary material for: Strengthened luteal phase support for patients with low serum progesterone on the day of frozen embryo transfer in artificial endometrial preparation cycles: a large-sample retrospective trial
Source: Reprod Biol Endocrinol. 2021 Apr 23;19:60. doi: 10.1186/s12958-021-00747-8 (PMC8063468; doi:10.1186/s12958-021-00747-8)
Supplement: Supplementary file 2 — Additional file 2: Supplemental Table 2 The pregnancy outcomes of artificial FET cycles in women without IVF failures. [file 12958_2021_747_MOESM2_ESM.docx]

**Supplemental table 2 The pregnancy outcomes of artificial FET cycles in women without IVF failures**

|  | Low P+ strengthened LPS  (Group A, n=1114) | Normal P+ routine LPS  (Group B, n=1105) | Crude RR (95%CI) | Adjusted RR (95%CI) | P value |
| --- | --- | --- | --- | --- | --- |
| Clinical pregnancy rate | 50.2% (559/1114) | 54.8% (606/1105) | 0.83(0.70,0.98) | 0.81(0.67,0.98) | 0.026 |
| Multiple pregnancy rate | 31.3% (172/550) | 27.7% (164/593) | 1.09(0.96,1.24) | 1.27(0.95,1.71) | 0.11 |
| Miscarriage rate | 16.2% (90/554) | 15.3% (91/594) | 1.07(0.78,1.47) | 1.04(0.73,1.48) | 0.83 |
| Live birth rate | 40.9% (456/1114) | 44.3% (490/1105) | 0.87(0.74,1.03) | 0.86(0.71,1.04) | 0.11 |

Adjusted for BMI, baseline FSH values, antral follicle counts, the number and stage of embryos transferred.
